# Cost-effectiveness of offering an area-level financial incentive on breast feeding: a within-cluster randomised controlled trial analysis

**DOI:** 10.1136/archdischild-2018-316741

**Published:** 2019-08-23

**Authors:** Nana Anokye, Kathryn Coyle, Clare Relton, Stephen Walters, Mark Strong, Julia Fox-Rushby

**Affiliations:** 1 Health Economics Research Group, Department of Clinical Sciences, College of Health and Life Sciences, Brunel University London, Uxbridge, UK; 2 School of Health and Related Research, University of Sheffield, Sheffield, UK; 3 Department of Population Health Sciences, Guy’s Campus, Kings College London, London, UK

**Keywords:** Health Economics, breast feeding, cost-effectiveness, RCT, financial incentive

## Abstract

**Objective:**

To provide the first estimate of the cost-effectiveness of financial incentive for breastfeeding intervention compared with usual care.

**Design:**

Within-cluster (‘ward’-level) randomised controlled trial cost-effectiveness analysis (trial registration number ISRCTN44898617).

**Setting:**

Five local authority districts in the North of England.

**Participants:**

5398 mother-infant dyads (intervention arm), 4612 mother-infant dyads (control arm).

**Interventions:**

Offering a financial incentive (over a 6-month period) on breast feeding to women living in areas with low breastfeeding prevalence (<40% at 6–8 weeks).

**Main outcome measures:**

Babies breast fed (receiving breastmilk) at 6–8 weeks, and cost per additional baby breast fed.

**Methods:**

Costs were compared with differences in area-level data on babies’ breast fed in order to estimate a cost per additional baby breast fed and the quality-adjusted life year (QALY) gains required over the lifetime of babies to justify intervention cost.

**Results:**

In the trial, the total cost of providing the intervention in 46 wards was £462 600, with an average cost per ward of £9989 and per baby of £91. At follow-up, area-level breastfeeding prevalence at 6–8 weeks was 31.7% (95% CI 29.4 to 34.0) in control areas and 37.9% (95% CI 35.0 to 40.8) in intervention areas. The adjusted difference between intervention and control was 5.7 percentage points (95% CI 2.7 to 8.6; p<0.001), resulting in 10 (95% CI 6 to 14) more additional babies breast fed in the intervention wards (39 vs 29). The cost per additional baby breast fed at 6–8 weeks was £974. At a cost per QALY threshold of £20 000 (recommended in England), an additional breastfed baby would need to show a QALY gain of 0.05 over their lifetime to justify the intervention cost. If decision makers are willing to pay £974 (or more) per additional baby breast fed at a QALY gain of 0.05, then this intervention could be cost-effective. Results were robust to sensitivity analyses.

**Conclusion:**

This study provides information to help inform public health guidance on breast feeding. To make the economic case unequivocal, evidence on the varied and long-term health benefits of breast feeding to both the baby and mother and the effectiveness of financial incentives for breastfeeding beyond 6–8 weeks is required.

**What is already known on this topic?**

- There is evidence that incentive-based breastfeeding programmes can increase breast feeding in areas with low rates.
- Solid evidence of the value for money of these programmes is lacking despite calls for such evidence.

**What this study adds?**

- This study reports, for the first time, cost-effectiveness estimates of the offer of a financial incentive for breast feeding in areas with low breastfeeding rates.
- This study provides new and high-quality data from a large cluster randomised controlled trial (with 92% follow-up data), with resource use data collected prospectively.
- Our study shows that these programmes can increase breast feeding and provide good value for money if decision makers are willing to pay £974 (or more) per additional baby receiving breastmilk.

## Introduction

Breast feeding has benefits for both mothers and babies.^1^ However, rates of any (ie, exclusive and mixed) breast feeding at age 12 months, are below 20%, on average, in high-income countries. The UK has the lowest rate (0.5%), Oman the highest (95%) and the USA has a rate of 27%.^2^ Even in low-income and middle-income countries with relatively higher breastfeeding rates at age 12 months, only 4 out of 10 babies younger than 6 months are exclusively breast fed.^2^ The low prevalence of breast feeding is estimated to cost high-income countries US$231 billion (0.5% of gross national income) annually.^1^ Policy makers in high-income countries are seeking effective and cost-effective interventions to encourage breastfeeding.^3^

Offering incentives to women to breast feed have been identified as an effective intervention to increase breast feeding and have been implemented in the USA,^4^ France^5^ and Canada.^6^ The first ever randomised controlled trial (RCT) of a financial incentive for breast feeding was conducted among 36 low-income Puerto Rican mothers who had initiated breast feeding. This US-based RCT found higher rates of continued breast feeding in the intervention group compared with control (89% vs 44% at 1 month; 89% vs 17%, at 3 months; 72% vs 0%, at 6 months).^4^ The authors recommended large-scale studies to assess clinical and cost-effectiveness of incentive-based breastfeeding interventions.

The first UK-based RCT of financial incentives for breast feeding was conducted as part of the Nourishing Start for Health (NOSH) project.^7^ This project developed and then trialled a structured population-level financial incentive for breastfeeding intervention that offered shopping vouchers to women if their infant was receiving breastmilk. The intervention was offered to all women living in areas with low breastfeeding prevalence (<40% at 6–8 weeks) in five local authority districts in the North of England. Up to five vouchers (£40 each) were offered to women if their baby was receiving breastmilk at the following ages: 2 days, 10 days, 6 weeks, 3 months and 6 months.

To date, no cost-effectiveness studies of financial incentives for breast feeding have been identified.^8^ However, review of breastfeeding incentive programmes by Moran *et al* 3 found eight studies with implementation costs data (but no cost-effectiveness estimates). To ensure the efficient allocation of resources in health systems, global and national public health decision makers need information on the value for money of these interventions. The WHO Breastfeeding Policy Brief^9^ identifies the need ‘to increase attention to, investment in and action for a set of cost-effective interventions and policies, that can help Member States and their partners’ to increase breast feeding and reach the WHO Global 2025 breastfeeding target of at least 50% of all infants being exclusively breastfed in the first 6 months.

This study, to the best of our knowledge examines, for the first time, the cost-effectiveness of offering an area-level financial incentive for breastfeeding intervention in a general population. Conducted alongside a large cluster (ward) RCT (trial registration number ISRCTN44898617), the analyses examined the within-trial cost-effectiveness of financial incentive for breast feeding in areas with low breastfeeding rates in the UK.

## Methods

The within-trial cost-effectiveness analysis compared the cost and benefits (in terms of babies receiving breastmilk) of offering financial incentives to women over a 6-month period postbirth versus control (no offer), from a healthcare provider perspective. The health outcome of interest was babies breast fed at 6–8 weeks and cost-effectiveness was reported as cost per additional baby breast fed over the four quarters of the 1-year trial. While data unavailability precluded estimating effectiveness at 6 months, total costs of vouchers were included in the analysis because the offer of vouchers up to 6 months was provided to participants at the outset of the trial and could therefore had impacted on the take up and duration of breast feeding. The protocol planned cost-effectiveness analysis^10^ was published prior to the analysis. The Trial Steering Committee approved changes from protocol to analyses. Changes were necessary because logistical and data constraints precluded: (a) the collection of area-level data on hospital admissions (related to gastrointestinal infection, otitis media, respiratory tract infections and atopic eczema) and (b) beyond trial modelling of the long-term cost-effectiveness of the intervention.

The total costs of providing the intervention in the trial included set up (website development, design and planning, booklet production, procurement, initial local engagement and staff induction) and delivery costs (including vouchers, processing of claims). Resource use data were extracted from trial management records, computer-based diaries and interviews with the trial manager. Resources were valued using national tariffs^11^ to increase generalisability. The unit costs of the vouchers were obtained from administrative records. Costs are expressed in pound sterling (2015–16), using the Hospital & Community Health Service inflation index where appropriate.^11^ As the trial was within 1 year, a discount rate was not applied.

Multivariable regression models adjusting for baseline variables and potential imbalances in treatment group were used to generate the incremental cost-effectiveness estimates.^12^ A generalised linear model using Poisson distributional family (and robust SEs) was fitted to generate the cost per ward/trial arm and incremental cost per ward. As the control areas had zero cost, a constant value of £0.001 was added to observations for model convergence. Cost per baby/trial arm was derived by dividing the estimated mean cost per ward by the number of babies per ward. A negative binomial model was used to estimate the intervention effect following the study by Relton *et al,* 7 although with different estimator. The outcome used in the study by Relton *et al*,^7^ percentage point increase in breastfeeding outcome, is a relative measure and not applicable to cost-effectiveness analysis.

To provide estimates of uncertainty, the ‘margins method’ generated sample means, by trial arm, for costs and breast feeding.^12^ The choice of distributional family for models was based on modified Park test^12^ and comparison of observed and predicted values. The covariates of the models included correlates of breastfeeding-related outcomes^7 13 14^: deprivation (Index Multiple Deprivation) score for the wards, baseline breastfeeding rate and ethnicity, and the inverse of the variance of breastfeeding rate (to account for the number of births in relation to breast feeding). The choice of covariates was based on a literature review conducted as part of this study to identify the potential predictors of breastfeeding-related outcomes.

Results are reported as cost per additional baby breast fed at 6–8 weeks. Deterministic sensitivity analyses assessed different components of total cost: (a) cost of routinely rolling out the scheme (covering induction and delivery costs) and (b) exclusion of the cost of voucher—this was to demonstrate the impact of assuming cost of vouchers is a ‘transfer payment’ (ie, giving women vouchers without any service in exchange); and therefore not includable in an economic evaluation. Probabilistic sensitivity analysis estimated the precision of the estimates of cost and breast feeding and investigated the robustness of potential differences in each. Bootstrap techniques (n=2000) based on regression models for costs and breastfeeding rates were employed to generate a sample of incremental costs and effects from an empirical distribution. This provided a measure of the probability that the intervention is cost-effective, at varying willingness to pay (WTP) values for changes in breastfeeding.

## Results

### Within-trial cost-effectiveness

Table 1 shows the descriptive statistics (unadjusted estimates). The total cost of providing the intervention in 46 wards, over 12 months and 5398 births in the period, was £462 600 with an average cost per ward of £9989 (SD £5538) and per baby of £91 (SD £22.40). Delivery costs constituted 86%, followed by recruitment (8%), set up (5%) and training (1%). The highest individual contributors were vouchers (74%; £342 840) and initial local engagement costs (4%; £19 598). Total cost per ward ranged from £2523 to £31 255 (online supplementary 1a–c). The control wards had no cost.

10.1136/archdischild-2018-316741.supp1 Supplementary data

**Table 1:** Average costs of intervention arm (pound sterling 2015–16)

Activities	Activities	Average cost per ward (SD) n=46	Average cost per baby (SD) n=5398
Set up	Preparation of booklets describing the scheme	£336 (0)	£2.86 (0)
	Design of intervention	£96 (0)	£0.82 (0)
	Development of the website with information about the scheme—including the postcode calculator	£64 (0)	£0.55 (0)
	Procurement of the vouchers from vendors (supermarkets and Love2shop)	£39 (0)	£0.33 (0)
	Initial local engagement	£426 (371)	£7.34 (9.17)
	Advertisement	£394 (0)	£3.36 (0)
	Training/Induction sessions for health visitors and midwives	£131 (70)	£1.65 (1.22)
Delivery	Vouchers	£7453 (5028)	£64.44 (18)
	Processing time for claim forms	£317 (214)	£2.74 (0.77)
	Information packs (including the booklets describing the scheme)	£283 (148)	£3 (1.46)
	Delivery of letters to mothers	£189 (122)	£2.10 (0.76)
	Costs of telephone, texts for processing claims	£166 (0)	£1.41 (0)
	Processing time for applications to join the NOSH scheme	£93 (60)	£0.84 (0.30)
**Total cost**		**£9989** (**5538**)	**£91.45** (**22.38**)

NOSH, Nourishing Start for Health.

Table 2 shows the regression-based estimates for costs, effects and incremental cost-effectiveness. Compared with control, the costs were higher for intervention wards (+£9738, 95% CI £8520 to £10 957). Online supplementary 2-3 show the regression-based estimates.

**Table 2:** Regression estimates for costs, effects and cost-effectiveness (pound sterling 2015–16)

	Control (46 wards; 4612 mother-infant dyads)		Intervention (46 wards; 5398 mother-infant dyads)	
	Mean	(95% CI)	Mean	(95% CI)
Base-case analysis				
Total cost per ward (£)	£0	(0 to 0)	£9738	(8520 to 10957)
Incremental cost (£)	–		£9738	(8520 to 10957)
Percentage of babies breast fed at at 6–8 weeks per ward	31.7%	(29.4 to 34.0)	37.9%	(35.0 to 40.8)
Total number of babies breast fed at 6–8 weeks per ward	29	(27 to 32)	39	(36 to 43)
Incremental number of breastfed babies	–		10	(6 to 14)
Cost per additional baby breast fed at 6–8 weeks (£)	–		£974	
Deterministic sensitivity analyses				
*Assuming that the provision of vouchers is the only accruable to the intervention*				
Total voucher cost per ward (£)	£0	(0 to 0)	£7251	(6117 to 8385)
Incremental non voucher cost (£)	–		£7251	(6117 to 8385)
Total number of babies breast fed at 6–8 weeks per ward	29	(27 to 32)	39	(36 to 43)
Incremental breast feeding	–		10	(6 to 14)
Voucher cost per additional baby breast fed at 6–8 weeks (£)	–		£725	
*Assuming that the provision of vouchers will be free of charge to the providers*				
Total non-voucher cost per ward (£)	£0	(0 to 0)	£2498	(2355 to 2638)
Incremental non-voucher cost (£)	–		£2498	(2355 to 2638)
Total number of babies breast fed at 6–8 weeks per ward	29	(27 to 32)	39	(36 to 43)
Incremental breast feeding	–		10	(6 to 14)
Non-voucher cost per additional baby breast fed at 6–8 weeks (£)	–		£250	
*Cost of routinely rolling out intervention*				
Total roll out cost per ward (£)	£0	(0 to 0)	£8402	(7154 to 9649)
Incremental roll out cost (£)	–		£8402	(7154 to 9649)
Total number of babies breast fed at 6–8 weeks per ward	29	(27 to 32)	39	(36 to 43)
Incremental breast feeding	–		10	(6 to 14)
Roll cost per additional baby breast fed at 6–8 weeks (£)			£840	

At baseline, area-level breastfeeding prevalence at 6–8 weeks was 27.4 (95% CI 25.2 to 29.6) in control and 28.7 (95% CI 26.7 to 30.6) in the intervention areas. At follow-up (for 1 April 2015 to 31 March 2016), area-level breastfeeding prevalence at 6–8 weeks was 31.7% (95% CI 29.4 to 34.0) in control areas and 37.9% (95% CI 35.0 to 40.8) in intervention areas.^7^ The adjusted difference between intervention and control was 5.7 percentage points (95% CI 2.7 to 8.6; p<0.001), resulting in 10 (95% CI 6 to 14) more additional babies breast fed in the intervention wards (39 vs 29). The cost per additional baby breast fed at 6–8 weeks was £974. Thus, at a cost per quality-adjusted life year (QALY threshold of £20 000 (recommended in England), an additional breastfed baby would need to show a lifetime total QALY gain to the infant and/or mother of 0.05 to justify the intervention cost. The required QALY gain decreases further to 0.03 if the threshold is £30 000.

**Figure 1:**
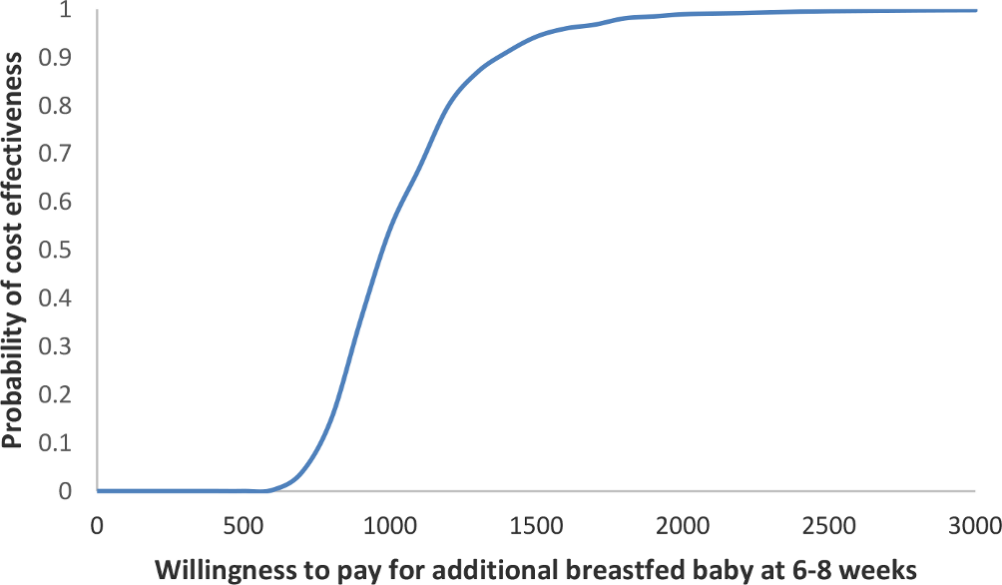
Cost effectiveness acceptability curve showing probability of cost effectiveness at different Willing to Pay (pounds sterling) values

Deterministic sensitivity analyses (table 2) show that voucher-only cost per additional baby breast fed at 6–8 weeks is £725 and £250 when only non-voucher costs are considered. Assuming the intervention is rolled out; the cost per additional baby breast fed will be £840. Probabilistic sensitivity analysis indicates that if decision makers’ WTP for additional breastfed baby is £1000, the intervention has 54% chance of being cost-effective (figure 1). At a WTP of £1500, the probability of intervention being cost-effective increases to 94% and to 99% if the WTP is £2000.

## Discussion

During the 1-year trial, the total cost of offering financial incentives for 5398 mother-infant dyads living in 46 areas with low breastfeeding prevalence was £462 600. Intervention areas compared with control required an additional cost (adjusted estimates) of £9738 (95% CI £8520 to £10,957) per ward, equivalent of £83 (95% CI £73 to £93) per baby. Compared with control areas, the intervention areas reported 10 more breastfed babies (95% CI 6 to 14) at 6–8 weeks per ward (39 vs 29). The mean cost per additional baby breast fed at 6–8 weeks was £974. There is a 54% chance of the scheme being considered cost-effective if decision makers were willing to pay £1000 per additional baby breast fed. Sensitivity analyses did not change this conclusion.

These findings feed into a sparse and mixed evidence base on cost-effectiveness of interventions to increase breast feeding.^15^ One UK study reported that breastfeeding groups facilitated by a health professional led to higher costs (£5 per attendance) and a lower breastfeeding rate at 6–8 weeks (−4%).^16^ Other studies showed that more intensive support and contact with health professionals offer good value for money with Rice *et al* 17 showing such interventions are cheaper and more effective. Hoddinott *et al* 18 compared the cost-effectiveness of team (proactive) and women-initiated (reactive) telephone support for breast feeding after discharge compared with reactive only and reported an incremental cost per additional woman breastfeeding of £87.

This is the first study to examine the cost-effectiveness of a financial incentive for breastfeeding intervention. The data on cost and effectiveness were sourced from a cluster RCT with 92% follow-up. The resource use data for costing were collected prospectively using mostly logging system and computer-based records. This method led to minimal errors with respect to ascertainment of resource use and no missing data, a rarity in trial-based economic evaluations.^19^

There are a number of limitations to this analysis. First, breast feeding has a wide range of benefits for both mothers and babies in both the short term and long term.^1^ This analysis did not account for data on health service use or utility estimates and this limits the comparison with non-breastfeeding programmes in the health sector. Second, the lack of data on the long-term benefits of breast feeding to both mother and child means that the value for money of the intervention is underestimated. Breast feeding has health benefits to both mothers and babies over the whole life course.^1^ Obtaining robust estimates of the life time costs and benefits of breast feeding to both the mother and baby is difficult due to the need to model outcomes far into the future, and was outside the scope of this analysis.

A 2012 comprehensive review by Renfrew *et al* found a clear association between increased breast feeding and reduced cases of necrotising entercolitis in preterm babies, acute otitis media, lower respiratory tract infections and gastrointestinal infections, which was of sufficient quality to allow the estimation of the economic impacts of improved breastfeeding rates.^20^ They showed that 45% of women exclusively breast feeding for 4 months and 75% of babies in neonatal units being breast fed at discharge can lead to 3285 fewer gastrointestinal infection-related admissions and 10 637 fewer general practitioner (GP) consultations (over £3.6 million treatment costs saving yearly); 5916 fewer lower respiratory tract infection-related hospital admissions and 22 248 fewer GP consultations (over £6.7 million treatment cost saving yearly); 21 045 fewer acute otitis media-related general practice consultations (over £750 000 treatment cost saving yearly) and 361 fewer cases of necrotising entercolitis (over £6 million treatment cost saving yearly). The application of these cost savings to the NOSH data is, however, challenged by the specific diseases included within the cost estimates. For example, the estimate on necrotising entercolitis was based on preterm babies within the neonatal intensive care unit (ICU). It would be inappropriate to attribute this cost savings to the increase in breast feeding within the NOSH trial, as the intervention was not targeted at mothers of preterm infants, and breastfeeding rates within the ICU were not assessed within the trial.

However, although evidence supported an association between increased breast feeding and improved cognitive outcomes, reduced early obesity and reduced sudden infant death syndrome, the available literature was not of sufficient quality to allow estimation of the scale and scope of the risk reduction with precision. With respect to the association between breast feeding and other diseases such as asthma, diabetes and cardiovascular disease, the strength of the evidence was also deemed not sufficient to allow estimation of the risk reduction and economic impact. Further well-designed studies are needed, which include adequate follow-up of outcomes, accurate definition and measurement of breast feeding and appropriate adjusting for confounding to inform the estimation of the long-term health and economic impacts of improved breast feeding.

With respect to quantifying how reasonable the 0.05 QALY gain over lifetime is (estimated to justify the intervention cost), with currently available data, this is difficult to do. The short-term benefits on acute otitis media, lower respiratory tract infection and gastrointestinal infections are generally associated with mild sequelae within the UK and are of limited duration, thereby resulting in only small utility deficits.^20–24^ On the other hand, some of the long-term sequelae, which do not have adequate data available currently to quantify accurately the relative risk reduction associated with breast feeding as listed above, would be associated with greater QALY deficits; however, many occur later in life and therefore the benefits and costs would be reduced due to discounting. Without an accurate estimate of the risk reduction associated with the increase in breast feeding achieved within the NOSH trial, it is not possible to model the impact on the incidence of long-term outcomes and consequently the potential QALY gain. Future studies are recommended to measure the short-term and long-term health impact of interventions.

Our analysis was based on the evidence from one trial, which tested a single permutation of the idea of offering financial incentives to mothers to breast feed. Future research is needed to help optimise this idea—testing a number of different variations. For example, would a universal single payment of £50 to mothers for exclusive breast feeding at 6–8 weeks be more or less effective in increasing breastfeeding rates? Additionally, the data on breast feeding were based on clinician reports collected as part of country-wide public health monitoring purposes. The validity of these reports are not usually assessed^7^ and therefore the use of objective measures of breastfeeding should be considered in future research.

This study provides information to help inform public health guidance on breast feeding. Implementing financial incentives to increase breast feeding in areas with low breastfeeding prevalence could offer value for money if policy makers are willing to pay £974 (or more) per additional baby breast fed. To make the economic case unequivocal, more research is required to provide effectiveness data on financial incentives for breast feeding beyond 6–8 weeks and epidemiological evidence on the varied health benefits of breast feeding to both the baby and mother. This will allow the incorporation of long-term health benefits of breast feeding in an economic analysis and facilitate the comparison of financial incentives for breast feeding with a wide range of other public health programmes and healthcare technologies.
