# Supplementary material for: Cost-effectiveness of offering an area-level financial incentive on breast feeding: a within-cluster randomised controlled trial analysis
Source: Arch Dis Child. 2019 Aug 23;105(2):155–9. doi: 10.1136/archdischild-2018-316741 (PMC7025724; doi:10.1136/archdischild-2018-316741)
Supplement: Supplementary data [file archdischild-2018-316741supp001.pdf]

Supplementary 1a: Average cost per baby by intervention ward

**Wards**			
	**Total average cost per baby per ward**	**No. of births**	**Total cost for ward**
**Derbyshire**			
Barrow Hill New Whittington	94.3	74	6976
Bolsover North West	101.1	58	5862
Clay Cross South	93.0	52	4837
Clowne South	117.7	30	3532
Hollingwood and Inkersall	81.3	80	6500
Killamarsh West	111.4	48	5347
Loundsley Green	71.6	37	2649
Lowgates and Woodthorpe	59.9	50	2994
Matlock St Giles	111.1	56	6224
Moor	75.8	42	3185
North Wingfield Central	74.1	69	5115
Old Whittington	119.7	39	4669
Pinxton	70.1	36	2525
Pleasley	168.9	18	3040
Scarcliffe	110.1	53	5836
Shirebrook Langwith	109.7	23	2523
South Normaton East	75.9	57	4326
**Sheffield**			
Firth Park	91.1	343	31255
Mosborough	115.3	151	17404
Richmond	85.6	176	15068
Southey	88.5	274	24253
Woodhouse	94.2	163	15347
**Rotherham**			
Anston & Woodsetts	85.5	74	6330
Boston Castle	103.5	190	19666
Holderness	84.2	128	10780
Hoober	92.2	150	13837
Rother Vale	100.9	131	13216
Rotherham East	79.4	233	18493
Silverwood	70.1	146	10239
Sitwell	118.7	104	12341
Wath	78.7	142	11180
Wingfield	62.0	109	6756
**Doncaster**			

**Wards**			
	**Total average cost per baby per ward**	**No. of births**	**Total cost for ward**
Armthorpe	85.5	180	15385
Askern Spa	80.5	119	9574
Bentley	74.7	216	16128
Central	61.5	316	19435
Great North Road	68.7	183	12579
Mexborough	59.7	238	14200
Sprotbrough	111.7	101	11280
Thorne	75.5	132	9964
Town Moor	68.2	173	11793
**Bassetlaw**			
East Retford East	124.6	77	9590
East Retford South	97.9	61	5973
Tuxford and Trent	136.8	43	5882
Worksop North	87.1	109	9498
Worksop North West	78.7	114	8977
**Mean (SD)**	91.45(22.38)	117.35 (78.49)	9989 (5538)

Supplementary 1b: Average cost per baby by intervention ward by activities

Wards							
	**Voucher**	**Info packs**	**Initial local engagement**	**Induction**	**Processing time for claims**	**Processing time for successful applications**	**Letters**
**Derbyshire**							
Barrow Hill New Whittington	66.5	2.0	9.7	1.5	2.8	0.8	1.6
Bolsover North West	69.7	2.6	12.4	1.9	3.0	0.8	1.6
Clay Cross South	60.0	2.9	13.8	2.1	2.6	0.8	1.6
Clowne South	69.3	5.0	23.9	3.6	3.0	1.2	2.4
Hollingwood and Inkersall	55.5	1.9	9.0	1.4	2.4	0.6	1.3
Killamarsh West	75.8	3.1	14.9	2.3	3.2	0.9	1.8
Loundsley Green	33.5	4.0	19.4	3.0	1.4	0.3	0.7
Lowgates and Woodthorpe	28.8	3.0	14.3	2.2	1.2	0.3	0.7
Matlock St Giles	78.6	2.7	12.8	2.0	3.3	0.8	1.7
Moor	40.0	3.5	17.1	2.6	1.7	0.5	1.1
North Wingfield Central	46.4	2.2	10.4	1.6	2.0	0.8	1.6
Old Whittington	79.0	3.8	18.4	2.8	3.4	1.0	2.0
Pinxton	31.1	4.1	19.9	3.0	1.3	0.4	0.9
Pleasley	97.8	8.3	39.8	6.1	4.2	1.1	2.3
Scarcliffe	76.2	2.8	13.5	2.1	3.2	1.0	2.0
Shirebrook Langwith	52.2	6.5	31.2	4.8	2.2	1.2	2.4
South Normaton East	45.6	2.6	12.6	1.9	1.9	0.6	1.3
**Sheffield**							
Firth Park	70.4	1.9	3.1	0.9	3.0	0.8	1.6
Mosborough	85.8	4.3	7.0	2.1	3.7	1.0	2.0
Richmond	59.8	3.7	6.0	1.8	2.5	0.8	1.7
Southey	66.7	2.4	3.9	1.2	2.8	0.7	1.5
Woodhouse	66.7	4.0	6.5	2.0	2.8	0.9	1.9
**Rotherham**							
Anston & Woodsetts	62.7	4.4	1.1	1.0	2.7	1.6	2.7
Boston Castle	84.0	1.7	0.4	0.4	3.6	0.5	3.6
Holderness	64.7	2.5	0.7	0.6	2.8	0.9	2.8
Hoober	72.8	2.2	0.6	0.5	3.1	0.7	3.1
Rother Vale	80.3	2.5	0.6	0.6	3.4	0.7	3.4
Rotherham East	62.1	1.4	0.4	0.3	2.6	0.5	2.6
Silverwood	52.1	2.2	0.6	0.5	2.2	1.0	2.2
Sitwell	95.8	3.1	0.8	0.7	4.1	0.8	4.1
Wath	60.0	2.3	0.6	0.5	2.6	0.9	2.6
Wingfield	42.9	3.0	0.8	0.7	1.8	1.6	1.8
**Doncaster**							

**Wards**							
	**Voucher**	**Info packs**	**Initial local engagement**	**Induction**	**Processing time for claims**	**Processing time for successful applications**	**Letters**
Armthorpe	67.1	1.6	0.5	0.6	2.9	0.6	2.9
Askern Spa	60.8	2.5	0.7	1.0	2.6	0.9	2.6
Bentley	57.6	1.4	0.4	0.5	2.5	0.6	2.5
Central	46.2	0.9	0.3	0.4	2.0	0.5	2.0
Great North Road	51.6	1.6	0.5	0.6	2.2	0.7	2.2
Mexborough	43.9	1.2	0.4	0.5	1.9	0.7	1.9
Sprotbrough	89.1	2.9	0.9	1.1	3.8	0.8	3.8
Thorne	56.7	2.2	0.7	0.9	2.4	0.9	2.4
Town Moor	50.9	1.7	0.5	0.7	2.2	0.8	2.2
**Bassetlaw**							
East Retford East	100.8	3.7	1.3	2.0	4.3	0.9	2.3
East Retford South	73.4	4.6	1.6	2.6	3.1	1.5	1.7
Tuxford and Trent	106.0	6.6	2.3	3.6	4.5	1.5	2.9
Worksop North	67.5	2.6	0.9	1.4	2.9	0.9	1.6
Worksop North West	59.6	2.5	0.9	1.4	2.5	1.0	1.5
**Mean (SD)**	64.44(18)	3(1.46)	7.34(9.17)	1.65(1.22)	2.7(0.77)	0.84(0.30)	2.10(0.76)

Supplementary 1c: Average cost per baby by intervention ward by activities

	Advertisement	Preparation of booklets	Website development	Telephone, texts for processing claims	Procurement	Design of intervention
Each ward (ie no variation)	3.4	2.9	0.6	1.4	0.3	0.8

Supplementary 2: Regression model estimates for babies breastfed at 6-8 weeks

**Independent variables**	Reduced model	
	Coef. [a]	*SE* [b]
**Trial arm**		
Reference category (control group)		
Intervention[c]	0.290***	0.062
**Location of residence**		
Reference category (Bassetlaw)		
Doncaster	0.101	0.137
North Derbyshire	-0.300**	0.113
Rotherham	0.489***	0.116
Sheffield	0.214	0.166
Baseline breastfeeding rate in ward	0.029***	0.006
Inverse of the variance of breastfeeding rate[d]	0.001***	0.000
No .of observations	92	
Constant	1.727***	
Pseudo R2	0.191	

[a] The estimated parameters and asterisks show significance level of 1%(***), 5%(**) , 10%(*)
[b] Standard error [c]The margins method was used to generate the incremental effects
[d] Included as covariate and not weights as a pragmatic option

Supplementary 3: Regression model estimates for costs of the intervention

**Independent variables**	Reduced model	
	Coef. [a]	*SE* [b]
**Trial arm**		
Reference category (control group)		
Intervention[c]	18.364***	0.060
Deprivation (IMD) score for ward	0.009*	0.005
Number of white people in ward	0.000***	0.000
Baseline breastfeeding rate in ward[d]	0.003	0.006
Inverse of the variance of breastfeeding rate[e]	0.000***	0.000
No .of observations	92	
Constant	-10.369***	

[a] The estimated parameters and asterisks show significance level of 1%(***), 5%(**) , 10%(*)
[b] Standard error [c]The margins method was used to generate the incremental costs
[d] Model fitness was better with its inclusion [e]Included as covariate and not weights as a pragmatic option

Anokye N, *et al. Arch Dis Child* 2020; 105:155–159. doi: 10.1136/archdischild-2018-316741
